# An Extended Kalman Filter and Back Propagation Neural Network Algorithm Positioning Method Based on Anti-lock Brake Sensor and Global Navigation Satellite System Information

**DOI:** 10.3390/s18092753

**Published:** 2018-08-21

**Authors:** Jie Hu, Zhongli Wu, Xiongzhen Qin, Huangzheng Geng, Zhangbin Gao

**Affiliations:** 1 Hubei Key Laboratory of Advanced Technology for Automotive Components, Wuhan University of Technology, Wuhan 430070, China; wuzhongli@whut.edu.cn; 2 Hubei Collaborative Innovation Center for Automotive Components Technology, Wuhan University of Technology, Wuhan 430070, China; 3 SAIC-GM-Wuling Automobile Co., Ltd., Liuzhou 545007, China; xiongzhen.qin@sgmw.com.cn (X.Q.); huangzheng.geng@sgmw.com.cn (H.G.); zhangbin.gao@sgmw.com.cn (Z.G.)

**Keywords:** ABS sensor, neural network, EKF, GNSS, T-Box

## Abstract

Telematics box (T-Box) chip-level Global Navigation Satellite System (GNSS) receiver modules usually suffer from GNSS information failure or noise in urban environments. In order to resolve this issue, this paper presents a real-time positioning method for Extended Kalman Filter (EKF) and Back Propagation Neural Network (BPNN) algorithms based on Antilock Brake System (ABS) sensor and GNSS information. Experiments were performed using an assembly in the vehicle with a T-Box. The T-Box firstly use automotive kinematical Pre-EKF to fuse the four wheel speed, yaw rate and steering wheel angle data from the ABS sensor to obtain a more accurate vehicle speed and heading angle velocity. In order to reduce the noise of the GNSS information, After-EKF fusion vehicle speed, heading angle velocity and GNSS data were used and low-noise positioning data were obtained. The heading angle speed error is extracted as target and part of low-noise positioning data were used as input for training a BPNN model. When the positioning is invalid, the well-trained BPNN corrected heading angle velocity output and vehicle speed add the synthesized relative displacement to the previous absolute position to realize a new position. With the data of high-precision real-time kinematic differential positioning equipment as the reference, the use of the dual EKF can reduce the noise range of GNSS information and concentrate good-positioning signals of the road within 5 m (i.e. the positioning status is valid). When the GNSS information was shielded (making the positioning status invalid), and the previous data was regarded as a training sample, it is found that the vehicle achieved 15 minutes position without GNSS information on the recycling line. The results indicated this new position method can reduce the vehicle positioning noise when GNSS information is valid and determine the position during long periods of invalid GNSS information.

## 1. Introduction

Along with the development of technologies such as cloud computing, big data and artificial intelligence, automotive technologies is being rapidly developed towards the new direction of “electronic, networking, intelligence and sharing” [1,2]. Telematics boxes (T-Boxes) [3] including functions such as positioning, long-distance communication and acquisition of vehicle status are being more popular. For example, the Chinese government requires new energy vehicles to be equipped with T-Boxes in accordance with the requirements of the Chinese GB-T32960 regulations to facilitate usage and management of new energy vehicles. Vehicle location information and status data may be collected by the T-Box. The vehicle information such as real-time locations, driving trajectories and operating status are networked to enterprise-provincial-national management platforms in accordance with requirements for individuals, governments, and enterprises to check them on the various management platforms.

The Global Navigation Satellite System (GNSS) is dominant in absolute positioning, However, due to costs limitation, the current T-Box positioning modules are low-cost, miniaturized, chip-level devices, vulnerable to highrise, overhead, tunnel, and other building shielding and multi-path effects [4,5], resulting in GNSS information with high noise and even positioning failures. Its disadvantages bring a lot of inconveniences. For example, the passengers cannot obtain the vehicle’s exact location and delay the travel time, the driver cannot perform real-time map navigation. Besides, in the time-sharing rental vehicle applications, the inaccurate vehicle location data cannot help passengers find the reserved vehicles well, and the leasing company cannot manage the vehicles well. 

How to improve the positioning precisions is a key research topic and difficulty in the vehicle position and navigation field. Researchers have proposed multiple integrated positioning methods. Some methods can improve the accuracy, and some even can satisfy autonomous vehicles’ high precision position needs [6]. Song et al. [7] in multiple Global Positioning System (GPS) information reception studies, proposed a positioning algorithm for multiple receivers to enhance positioning performance in urban areas. Lu et al. [8] proposed a direct position determination (DPD) algorithm that is superior in terms of high estimation accuracy and strong resolution capability. Zhang et al. [9,10,11,12] enhanced the vehicle position precision by integrating a high precision inertial navigation system, particle filtering and Kalman filtering. Malleswaran et al. [13,14,15,16] extended inertial navigation’s stable position time through a deep learning algorithm to reduce devices’ cumulative error. However, this method is limited by the need for expensive inertial devices. Quddus M A et al. [17,18,19] presented a map-matching algorithm to correct vehicle positions, but such an algorithm is limited because many roads are not marked on the map. Alam et al. [20,21,22,23,24] put forward a Roadside Unit (RSU)-based method to improve the position precision, but RSUs have not yet been popularized. Melendez-Pasto et al. [25,26] proposed improved position accuracy measures by integrating an Extended Kalman Filter (EKF) based on Antilock Brake System (ABS) sensor data. However, this method didn’t address the positioning problem when GNSS information is invalid. Trehard et al. [27,28,29] applied Robot Simultaneous Localization and Mapping (SLAM) technology to vehicles to solve partial area planned roads’ position matching problems but the road data in maps is too large and complex. To improve the position accuracy and solve the positioning problem when GNSS information is invalid, this paper proposes a GNSS data real-time fusion method based on ABS sensor data. A new positioning method based on ABS sensors and GNSS information fusion was put forward, where communication was performed by the CAN bus and onboard diagnosis (OBD) system [30]. The T-Box acquires data of from ABS sensors and the GNSS positioning information of positioning modules. Then, data were uploaded from the T-Box to a server for processing, for which the EKF algorithm was utilized to reduce the GNSS information error and the Back Propagation Neural Network (BPNN) algorithm was applied to solve the positioning inability in the case of GNSS positioning invalidity.

The structure of this paper is as follows: the next section mainly discusses the structure of the T-Box system and the specific process of the fusion positioning method. The third section is based on the dual EKF research, including getting the state and measurement of the Pre-EKF and After-EKF. The fourth section is the BPNN model analysis, including the processing of input and output normalization. The fifth section experimentally verifies the feasibility of dual EKF filtering for roads with good-signal positioning signals and the verification of BPNN on the circulation route by artificially shielding the positioning signal. The sixth section presents the conclusions and outlook, which summarizes the contents of this paper and presents the deficiencies of this paper.

## 2. Positioning Scheme Based on ABS Sensor and GNSS Information Fusion

### 2.1. Fusion Positioning System

**Figure 1:**
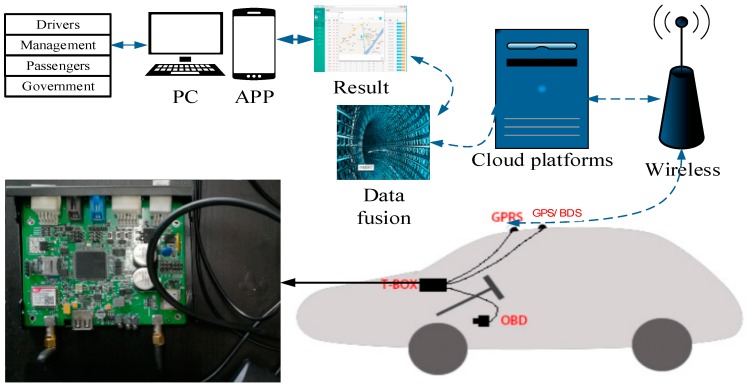
Fusion positioning system architecture.

The positioning system of this paper is a typical terminal-management-cloud structure shown in Figure 1. It is made up of three parts: a T-Box, wireless communication and cloud platforms. The T-Box primarily includes a positioning module, a remote communication module, controller area network (CAN) communication module and a microprocessor. The T-Box acquires ABS data through the CAN communication module and gets GNSS data from a Beidou positioning dual system (BDS) (containing GPS and Beidou units) and then send the above data to the cloud platform through the wireless communication platform. The ABS sensor data and GNSS data are fused on the cloud platform by means of dual EKF and BPNN and then there is a real-time position estimation. The platform sends the information to users (such as drivers, management, passengers, government and so on), thus, the system can effectively improve the position accuracy and enhance users’ experience. 

### 2.2. Fusion Positioning Method

**Figure 2:**
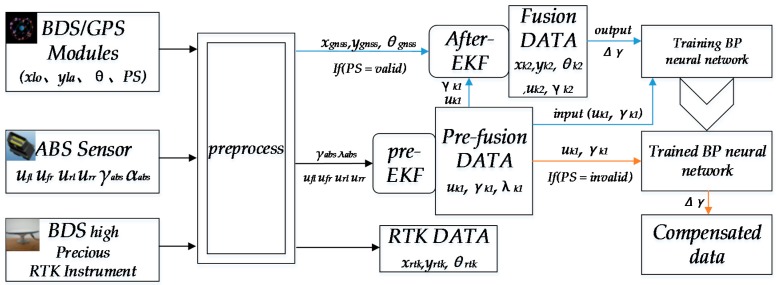
The position method structure.

The positioning method structure is shown as Figure 2. The nomenclature used in the paper is listed in Table 1. The preprocessing phase includes three steps. First, convert wheel angles from the ABS sensor into *λ_abs_* values. Second, judge the positioning validity status. Third take the vehicle’s starting position as the basic point, convert *x_lo_*, *y_la_* to absolute position *x_gnss_* (m), *y_gnss_* (m) and convert the high-precision RTK differential position data into the absolute position values *x_rtk_*, *y_rtk_*. The fusion position algorithm involves EKF and BPNN. The EKF is a nonlinear version of the Kalman filter which linearizes about an estimate of the current mean and covariance.

In this paper, the EKF algorithm is used to reduce the error of the Gaussian distribution of the ABS and GNSS data in the nonlinear system when the GNSS positioning status is valid. Nomenclature related to the method are presented in Table 1. When the GNSS positioning status is invalid or invalid for a long time, ABS fused data *u_k_*_1_, *γ_k_*_1_ can be synthesized from the relative position, but this positioning method is easily suffers from interference from heading angle speed errors, resulting relative positioning failure. The purpose of BPNN is to obtain the heading angle speed error corresponding to different vehicle and heading angle speeds. The BPNN network is a kind of multilayer feed forward network with the error back propagation nature. There is no need to set up an initial dynamic or noise model and it will find a relationship among Δ*γ* and *u_k_*_1_, *γ_k_*_1_ through self-study. The concrete steps of the fusion position method are as follows: fuse ABS sensor data *u_fl_*, *u_fr_*, *u_rl_*, *u_rr_*, *γ_abs_* and *λ_abs_* to *u_k1_* and *γ_k_*_1_ by Pre-EKF. Distinguish positioning valid status, if the status is valid, through After-EKF fuse *u_k_*_1_, *γ_k_*_1_ and GNSS data *x_gnss_*, *y_gnss_*, *θ_gnss_* to the new positioning data *x_k_*_2_, *y_k_*_2_ and *θ_k_*_2_. In the BPNN structure, the training sample output value Δ*γ* is from *θ_k_*_2_ and *γ_k_*_1_. *u_k_*_1_ and *γ_k_*_1_ serve as input values of the training samples *to* train the BPNN. When the GNSS positioning status is invalid, we can put *u_k_*_1_ and *γ_k_*_1_ into the well-trained BPNN and get Δ*γ*. The relative location is synthesized through *u_k_*_1_ with a corrected *γ_k_*_1_ by Δ*γ* in accordance with the dead reckoning method [31]. 

## 3. Dual Kalman Filtering-Based Positioning Research

Dual Extended Kalman Filter respectively refers to Pre-EKF (based on a vehicle kinematical model) and After-EKF. The purpose of Pre-EKF is to fuse the ABS data’s four-wheel speed, yaw rate and steering wheel angle to determine an accurate vehicle speed and heading angle velocity. After-EKF is used to reduce the noise of GNSS data by fusing *u_k_*_1_, *γ_k_*_1_ and GNSS data. The purpose of the dual EKF design is to improve the position accuracy, and provide low-noise training and validation samples for the BP neural network algorithm. And nomenclature related to the structure of the car are presented in Table 2.

### 3.1. Fusion Positioning System

**Figure 3:**
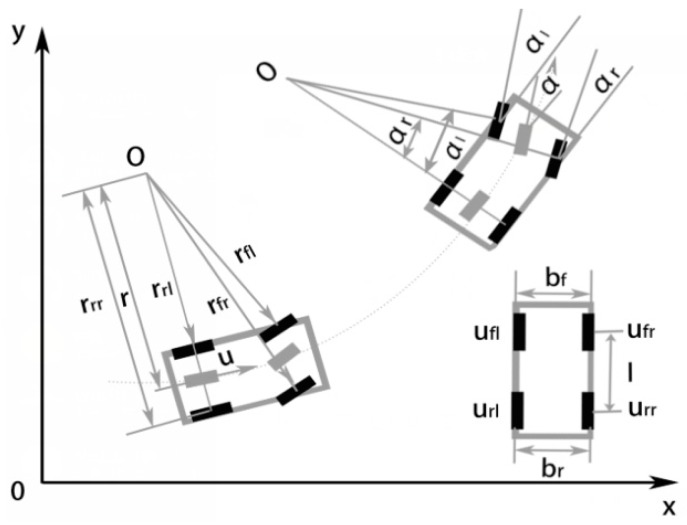
Vehicle turning schematic diagram.

The establishment of the Pre-EKF equations is by vehicle kinematics analysis. While a vehicle is turning to prevent any additional resistance between vehicle and road and excessive tire wear, the design of the complete vehicle steering mechanism requires conformance to the Ackerman Principle [32]. As for a vehicle turning at a certain speed (Figure 3), all wheels are assumed to perform pure rolling motion, and the axes of all wheels intersect at the turning center (Point O) at this time. The complete vehicle may be virtually simplified as a two-wheel motorcycle model [33] with two degrees of freedom. In addition, the virtual steering angle corresponds to the center of the front axle. The following Equations (1)–(9) are quoted from [25], which gives a detailed introduction to the derivation process. This paper is slightly improved on this basis, where λ = *tan* (α) is assumed to simplify the calculation of the EKF algorithm. 

In accordance with the turning principle and the assumption of the virtual steering angle, the turn radii of four wheels are expressed by:

(1) $$r=\frac{l}{\lambda }$$

(2) $$r_{fl}=\sqrt{{\left(\frac{l}{\lambda }-\frac{b_{f}}{2}\right)}^{2}+l^{2}}$$

(3) $$r_{fr}=\sqrt{{\left(\frac{l}{\lambda }+\frac{b_{f}}{2}\right)}^{2}+l^{2}}$$

(4) $$r_{rl}=\frac{l}{\lambda }-\frac{b_{r}}{2}$$

(5) $$r_{rr}=\frac{l}{\lambda }+\frac{b_{r}}{2}$$

Speeds of the four wheels are calculated by:

(6) $$u_{fl}=\frac{u}{r}\cdot r_{fl}=u\times \sqrt{\lambda^{2}+{\left(1-\frac{\lambda \cdot b_{f}}{2l}\right)}^{2}}$$

(7) $$u_{fr}=\frac{u}{r}\cdot r_{fr}=u\times \sqrt{\lambda^{2}+{\left(1+\frac{\lambda \cdot b_{f}}{2l}\right)}^{2}}$$

(8) $$u_{rl}=\frac{u}{r}\cdot r_{rl}=u\cdot (1-\frac{\lambda \cdot b_{r}}{2l})$$

(9) $$u_{rr}=\frac{u}{r}\cdot r_{rr}=u\cdot (1+\frac{\lambda \cdot b_{r}}{2l})$$

The relationship between λ, *u* and γ is expressed as:

(10) $$\lambda =\frac{l}{u}\cdot \gamma$$

As for designing variables for the EKF state equations, the relative motion of the vehicle may be derived from u and γ. On the other hand, with a view to α being greatly related to $u_{k_{1}}$ and $\gamma_{k_{1}}$ and the whole state equation of the entire Pre-EKF system including three variables ($u_{k_{1}}$, $\gamma_{k_{1}}$ and $\lambda_{k_{1}}$), the corresponding state equation is as follows:

(11) $$X_{1\;k_{1}}={\left(u_{k_{1}},\gamma_{k_{1}},\lambda_{k_{1}}\right)}^{T}\;\begin{array}{l} =f_{1}\left(X_{1k_{1}-1}\right)+{W_{1}}_{k_{1}-1}\\ =\left(\begin{array}{c} u_{k_{1}-1}\\ \gamma_{k_{1}-1}\\ \lambda_{k_{1}-1} \end{array}\right)+{W_{1}}_{k_{1}-1} \end{array}$$

The variables of the measurement equation include $u_{fl}$, $u_{fr}$, $u_{rl}$, $u_{rr}$, $\gamma_{abs}$ and $\lambda_{abs}$ (to be measured and converted by the steering wheel angle sensor). In accordance with the above kinematics analysis of a turning vehicle, the system measurement equation is written as:

(12) $$\begin{array}{cc} {Z_{1}}_{k_{1}} & =h_{1}(X_{1k_{1}})+V_{1k_{1}}\\ & ={\left(u_{fl},u_{fr},u_{rl},u_{rr},\lambda_{\mathrm{abs}},\gamma_{abs}\right)}^{T}+{V_{1}}_{k_{1}}\\ & \begin{array}{l} =\left(\begin{array}{l} u_{k_{1}}\cdot \sqrt{\lambda^{2}+{\left(1+\frac{\lambda \cdot b_{f}}{2l}\right)}^{2}}\\ u_{k_{1}}\cdot \sqrt{\lambda^{2}+{\left(1-\frac{\lambda_{k_{1}}\cdot b_{f}}{2l}\right)}^{2}}\\ u_{k_{1}}\cdot (1+\frac{\lambda_{k_{1}}\cdot b_{r}}{2l})\\ u_{k_{1}}\cdot (1-\frac{\lambda_{k_{1}}\cdot b_{r}}{2l})\\ \gamma_{k_{1}}\cdot \frac{l}{u_{k_{1}}}\\ \gamma_{k_{1}} \end{array}\right)+V_{1k_{1}} \end{array} \end{array}$$

where ***W*_1_** **~*N* (0, *Q*_1_)** is the state noise, following a Gauss distribution with zero vector mean and covariance matrix ***Q*_1_**, ***V*_1_** **~*N* (0, *R*_1_)** is the measurement noise, following a Gauss distribution with zero vector mean and covariance matrix ***R*_1_**. 

### 3.2. After-EKF Model

In case the GNSS information to the positioning module is valid and its $x_{k_{2}}$, $y_{k_{2}}$, $\theta_{k_{2}}$, $u_{k_{2}}$ and $\gamma_{k_{2}}$ are selected as the variables of the state equation of the After-EKF, which is expressed as:

(13) $${X_{2}}_{2k_{2}}={\left(x_{k_{2}},y_{k_{2}},\theta_{k_{2}},u_{k_{2}},\gamma_{k_{2}}\right)}^{T}\;\begin{array}{l} =f_{2}\left(X_{2k_{2}-1}\right)+W_{2k_{2}-1}\\ =\left(\begin{array}{c} x_{k_{2}-1}+u_{k_{2}-1}\cdot \cos(\theta_{k_{2}-1})\cdot \Delta t\\ y_{k_{2}-1}+u_{k_{2}-1}\cdot \sin(\theta_{k_{2}-1})\cdot \Delta t\\ \theta_{k_{2}-1}+\gamma_{k_{2}-1}\cdot \Delta t\\ u_{k_{2}-1}\\ \gamma_{k_{2}-1} \end{array}\right)+W_{2k_{2}-1} \end{array}$$

where Δ*t* is data fusion interval. $u_{k_{1}}$ and $\gamma_{k_{1}}$ obtained by the Pre-EKF algorithm based on the vehicle kinematics fusion filtering. 

Heading angle $\theta_{gnss}$, relative latitude-conversion $x_{gnss}$ and relative longitude-conversion $y_{gnss}$ obtained by the chip-level GNSS receiving module, serve as variables of the measurement equation for the After-EKF filtering system, which is expressed as:

(14) $$Z_{2k_{2}}=\left(x_{gnss},y_{gnss},\theta_{gnss},u_{k_{1}},\gamma_{k_{1}}\right)\;Z_{2k_{2}}=h_{2}(X_{2k_{2}})+V_{k_{2}}\;={\left(x_{k_{2}},y_{k_{2}},\theta_{k_{2}},u_{k_{2}},\gamma_{k_{2}}\right)}^{T}+V_{k_{2}}$$

where ***W_2_*** **~*N* (0, *Q*_2_)** is the state noise, following a Gauss distribution with zero vector mean and covariance matrix ***Q_2_, V*_2_** **~*N* (0, *R*_2_)** is the measurement noise, following a Gauss distribution with zero vector mean and covariance matrix ***R*_2_**. 

The EKF is a first-order linearization truncation to the Taylor expansion of the nonlinear functions *f(∙)* and *h(∙)* and neglects the other higher order terms. The Jacobian determinant of EKF state matrix ***F_k_*** and measurement matrix ***H_k_*** can be derived by the derivatives of the functions *f(∙)* and *h(∙).* The results from such calculations are provided in Equations (15)–(18) respectively:

(15) $$F_{1k}=\frac{\partial f_{1}}{\partial x_{1k}}=\left(\begin{array}{ccc} 1 & 0 & 0\\ 0 & 1 & 0\\ 0 & 0 & 1 \end{array}\right)$$

(16) $$H_{1k}=\frac{\partial h_{1}}{\partial x_{1k}}=(\begin{array}{ccc} \sqrt{{\left(1+\frac{b_{r}}{2\cdot l}\cdot \lambda_{k1}\right)}^{2}+{\lambda_{k1}}^{2})} & 0 & \frac{u_{k1}\cdot (2\cdot l+b_{f}\cdot (1+\frac{b_{f}}{2\cdot l\cdot \lambda_{k1}}))}{\sqrt{\left({\left(\frac{2l}{\lambda_{k1}}+b_{f}\right)}^{2}+4\cdot l^{2}\right)}}\\ \sqrt{{\left(1-\frac{b_{r}}{2\cdot l}\cdot \lambda_{k1}\right)}^{2}+{\lambda_{k1}}^{2})} & 0 & \frac{u_{k1}\cdot (2\cdot l-b_{f}\cdot (1-\frac{b_{f}}{2\cdot l\cdot \lambda_{k1}}))}{\sqrt{\left({\left(\frac{2\cdot l}{\lambda_{k1}}-b_{f}\right)}^{2}+4\cdot l^{2}\right)}}\\ \frac{\left(2\cdot l+b_{r}\cdot \lambda_{k1}\right)}{2\cdot l} & 0 & \frac{\left(b_{r}\cdot u_{k1}\right)}{2\cdot l}\\ \frac{\left(2\cdot l-b_{r}\cdot \lambda_{k1}\right)}{2\cdot l} & 0 & -\frac{\left(b_{r}\cdot u_{k1}\right)}{2\cdot l}\\ -\frac{l}{{u_{k1}}^{2}}\cdot \gamma_{k1} & \frac{l}{u_{k1}} & 0\\ 0 & 1 & 0 \end{array})$$

(17) $$F_{2k}=\frac{\partial f_{2}}{\partial x_{2k}}=\left(\begin{array}{ccccc} 1 & 0 & -u_{k2-1}\cdot \sin(\theta_{k2-1})\cdot \Delta t & \cos(\theta_{k2-1})\cdot \Delta t & 0\\ 0 & 1 & u_{k2-1}\cdot \cos(\theta_{k2-1})\cdot \Delta t & \sin(\theta_{k2-1})\cdot \Delta t & 0\\ 0 & 0 & 1 & 0 & \Delta t\\ 0 & 0 & 0 & 1 & 0\\ 0 & 0 & 0 & 0 & 1 \end{array}\right)$$

(18) $$H_{2k}=\frac{\partial h_{2}}{\partial x_{2k}}=\left(\begin{array}{ccccc} 1 & 0 & 0 & 0 & 0\\ 0 & 1 & 0 & 0 & 0\\ 0 & 0 & 1 & 0 & 0\\ 0 & 0 & 0 & 1 & 0\\ 0 & 0 & 0 & 0 & 1 \end{array}\right)$$

## 4. Study of Positioning Method Based on BP Neural Network

**Figure 4:**
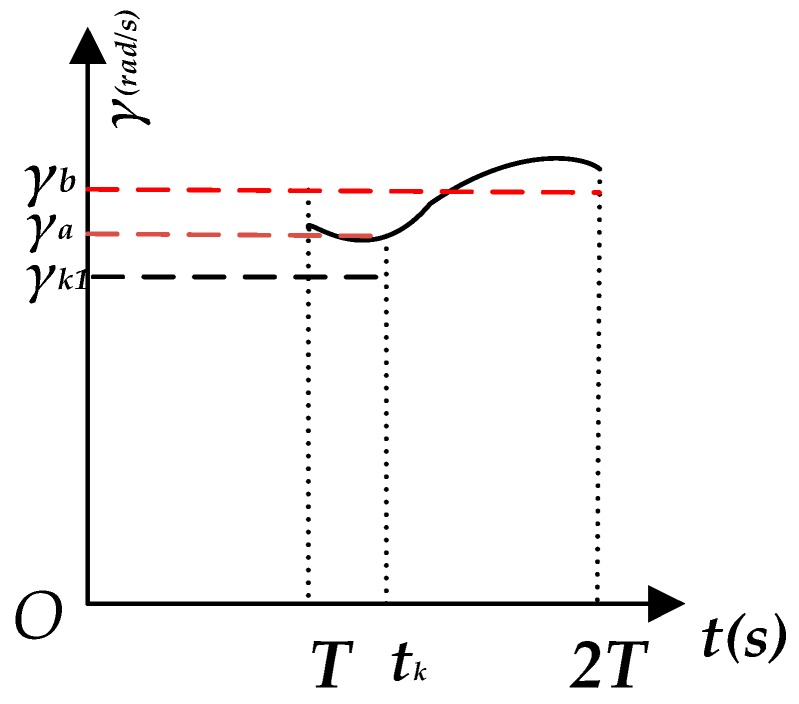
*γ* (rad/s) versus time *t* (s).

The study of the positioning method based on the BP neural network is primarily to solve the positioning issue and achieve the long-term positioning in the case invalid of GNSS positioning with the help of data from ABS. $u_{k_{1}}$ and $\gamma_{k_{1}}$ may be obtained by the Pre-EKF fusion and the θ can be obtained by integration of $\gamma_{k_{1}}$ with time. The dead reckoning algorithm indicates that the vehicle position information in case of invalid GNSS positioning may be obtained based on $u_{k_{1}}$ and θ. The error of θ will gradually increase as the error of $\gamma_{k_{1}}$ accumulates. It is assumed that the actual change of γ within a period (*T*) is as shown in Figure 4. The meaning of the two parameters in Figure 4 is as follows:

$\gamma_{a}$: actual value of γ corresponding to time $t_{k}$
$\gamma_{b}$: expected value referring to the average value of γ from time *T* to time 2*T*

Measurement errors exist for $\gamma_{k_{1}}$ and $\gamma_{a}$ due to the fact that ABS data may be affected by factors such as temperature, assembly and measurement precision. Moreover, the difference between $\gamma_{k_{1}}$ and $\gamma_{b}$ is finally relatively large because the acquisition cycle time *T* is relatively long, and γ changes more frequently when the car turns sharply. $\gamma_{k_{1}}$ is corrected based on the BP neural network model to reduce the difference between $\gamma_{k_{1}}$ and $\gamma_{b}$. Assuming Δ*γ* as the deviation of $\gamma_{k_{1}}$ and $\gamma_{b}$, it is known based on analysis of γ changing with time, that there exists a non-linear relationship between Δ*γ*, and $u_{k}$ and $\gamma_{k_{1}}$, which may be expressed by construction of the BP neural network model for two input layers and one output layer. 

The scheme of this paper is to train the BPNN algorithm by using the heading angle speed error *γ* calculated from Equation (19) as the output target data (note: *γ* sourced from GNSS information is not an ideal datapoint, there is certain noise, but it is relatively small compared to the ABS acquisition of information noise). In order to prevent BPNN from fitting the linearity, cross validation is used to solve the problem of overfitting. The BPNN training time is relatively long, but with the help of the high computing speed of the cloud platform, the real-time performance is improved. 

### 4.1. BP Neural Network Model

**Figure 5:**
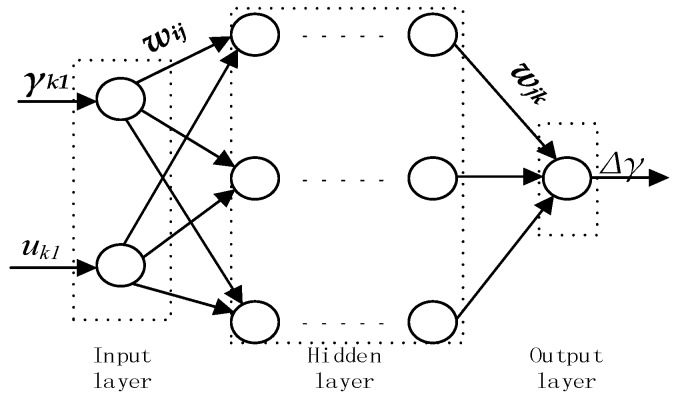
BP neural network structure diagram.

The structural diagram of the BP neural network is shown in Figure 5. Input signals are in forward propagation based on the sequence (input layer → hidden layer → output layer). Signals are sent from each node to all nodes. If the expected output is not obtained during the training process, the error will be predicted by means of the BP neural network to adjust the network weight and threshold.

The BPNN has a strong non-linear fitting ability. When the GNSS positioning status is valid, $\gamma_{k_{1}}$ and $u_{k_{1}}$ after the Pre-EKF data fusion are taken as the input variables and Δ*γ* calculated by Equation (19) based on data gained after the After-EKF data fusion is taken as the output target so that samples may be trained by the BP neural network algorithm:

(19) $$\Delta \gamma =\frac{\left(\theta_{k_{2}}-\theta_{k_{2}-1}\right)}{\Delta t}-\gamma_{k_{1}}$$

### 4.2. Determination of the BP Neural Network Structure

For elimination of any adverse effect due to large differences between variables, the characteristic parameters ($\gamma_{k_{1}}$, $u_{k_{1}}$ and Δ*γ*) of the system are normalized based on Equation (20) so that they shall be distributed in [−1, 1]:

(20) $$a_{i}=\frac{2A_{i}-(A_{\max}+A_{\min})}{\left(A_{\max}-A_{\min}\right)}$$

where *A_i_* and *a_i_* represent the original and normalized datum, respectively, *A_max_* and *A_min_* represent the maximum and minimum of *A_i_*, respectively. 

The number of hidden layer(s) and the number of nodes in the hidden layer are determined by repeated calculation. The number of hidden layer(s) starts from 1, and when good results are not achieved in this case, the number of hidden layer(s) may be added. The number of nodes in the hidden layer may be determined based on the following empirical formula:

(21) $$p=\sqrt{n+m}+q$$

where *p* represents the number of nodes in the hidden layer, *m* and *n* represent the numbers of input and output variables, respectively, and q represents an integer between 1 and 10. 

The number of hidden layers is selected as two in the neural network and three nodes per hidden layer, which is the result of applying the BP neural network model many times. The transfer function for any node in the hidden layer is the tangent S type and a linear transfer function is selected for the output layer.

## 5. Experimental Study and Analysis of Results

### 5.1. Testing Program

For verifying the positioning precision effects of the T-Box of the dual EKF system and the duration of reliable positioning after correcting the heading angle speed by the BP neural network algorithm in the cases where GNSS positioning status is invalid, a corresponding outdoor vehicle field test plan was designed to perform an experimental analysis. Throughout the experiment, positioning data received by a Beidou high-precision RTK differential positioning units acted as the high-precision reference data. The GNSS receiver module is a chip-level BD/GPS dual-mode satellite navigation receiver module embedded in an onboard device, which selects an effective signal as positioning data. The data acquisition frequency of the onboard GNSS receiving module is 1 Hz. The data acquisition frequency of the onboard ABS sensor is 5 Hz. The Beidou differential positioning system is connected to a computer whose sampling differential positioning data frequency is 5 Hz. ABS data are collected in accordance with ISO15765 protocol (auto diagnosis protocol) by the OBD interface of the T-Box, and GNSS data are sent from the positioning modules to the cloud server by the remote communication module, where the received data are stored in the data library.

**Figure 6:**
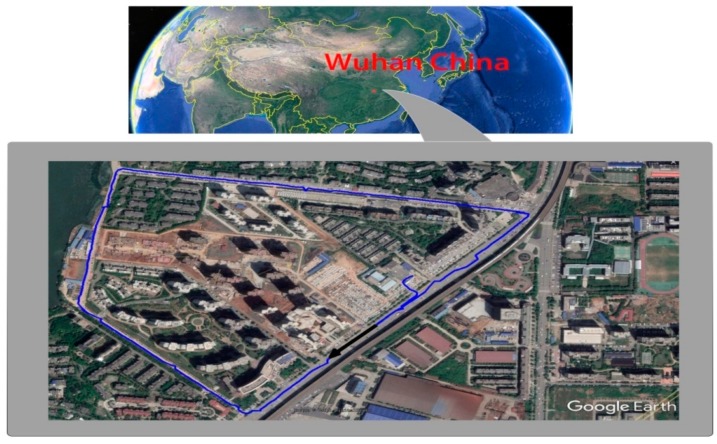
Vehicle driving trajectory map.

The basic parameters of the test vehicle are show in Table 3. The test area is a road (width: about 5 m) in Wuhan, where valid GNSS positioning is very good. A part of the driving trajectory is shown in Figure 6.

The driving test continued for up to 50 min. The original longitude and latitude data from the T-Box and the corresponding data from the ABS sensors are preliminarily processed on the PC PC to generate 45 min of effective GNSS data. Fusion is carried out first with the data of the ABS sensors by use of the Pre-EKF algorithm, and the ***Q*_1_** and ***R*_1_** values of the Pre-EKF covariance matrix are adjusted to achieve the optimal filtering effect. 

### 5.2. Analysis of Positioning Effects in Case of GNSS Positioning Status Being Valid

Fusion is carried out to $x_{gnss}$, $y_{gnss}$, and $\theta_{gnss}$ acquired by the low cost BDS/GPS dual-mode satellite navigation unit by use of the After-EKF filtering algorithm and $u_{k_{1}}$ and $\gamma_{k_{1}}$ obtained by use of the Pre-EKF algorithm, and the ***Q*_2_** and ***R*_2_** values of the After-EKF covariance matrix are adjusted to achieve the optimal filtering effect. 

While the navigation error distance is assumed as *D*, and $x_{RTK}$ and $y_{RTK}$ represent the converted latitude and longitude data of the Beidou differential positioning system; their relationship is expressed as:

(22) $$D=\sqrt{{\left(x-x_{RTK}\right)}^{2}+{\left(y-y_{RTK}\right)}^{2}}$$

**Figure 7:**
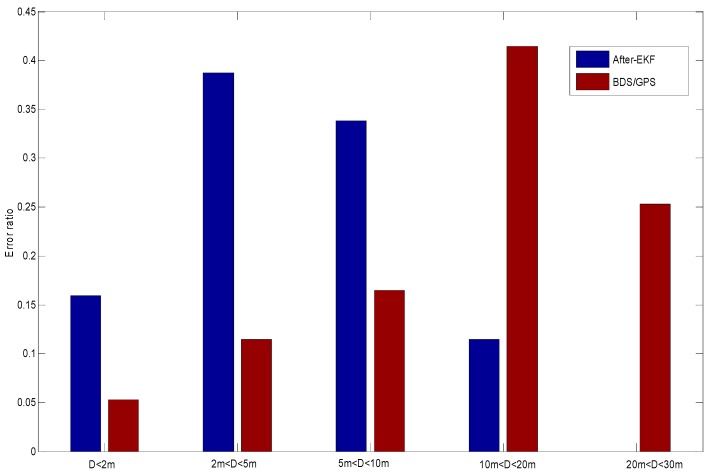
Vehicle positioning error distance distribution map.

With reference to the high-precision reference positioning data, a comparison is carried out to the original latitude and longitude obtained by the GNSS receiving module and the latitude and longitude data processed by means of the dual EKF model, and the distribution of their navigation error ratios is shown in Figure 7.

*D* for the original positioning data is up to above 40 m, while the navigation error for the data corrected by the dual EKF algorithm may be effectively controlled within 18 m. Analysis of the results shown in Figure 7 indicates that coverage errors are above 10 m for most of the original positioning data, but the processed coverage errors are within 10 m. Thus, the positioning precision may be effectively improved and the errors of the low-cost positioning module may be actually reduced by means of the dual EKF processing of ABS data. 

### 5.3. Analysis of Positioning Effects in Case of Invalid of GNSS Positioning Status

45 min of vehicle test data are processed by means of the dual EKF algorithm to extract the data ($u_{k_{1}}$, $\gamma_{k_{1}}$ and Δ*γ*). The 45 min vehicle test data are divided into two groups based on time:

(1) 30 min sample data;
(2) 15 min performance display data.

**Figure 8:**
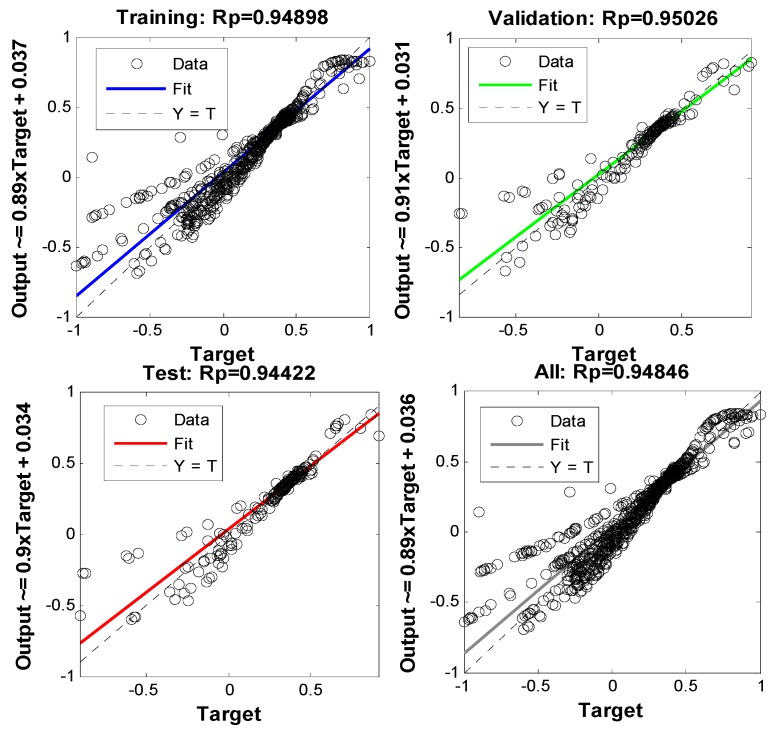
BP neural network linear regression results.

In order to prevent overfitting during BPNN training, the 30 min sample data are divided into the training, the verification and the test data. Training data are primarily to train the neural network model; and the verification and measurement data are primarily utilized to verify the training effects of the BP neural network. The regression precision analysis of the BP neural network model is included. The linear regression results of the training, verification and measurement results are shown in Figure 8 (“target” means the value normalized Δ*γ*, “output” means $u_{k_{1}}$ and $\gamma_{k_{1}}$ input to the well-trained BPNN output value). 

The linear regression results indicate that the errors of the training, verification and test results are randomly distributed on the both positive and negative sides, and the correlation coefficient (Rp) of the predicted and measured results of the training, verification and test samples is more than 0.94, thus, the nonlinear fitting degree of the model is very high. 

**Figure 9:**
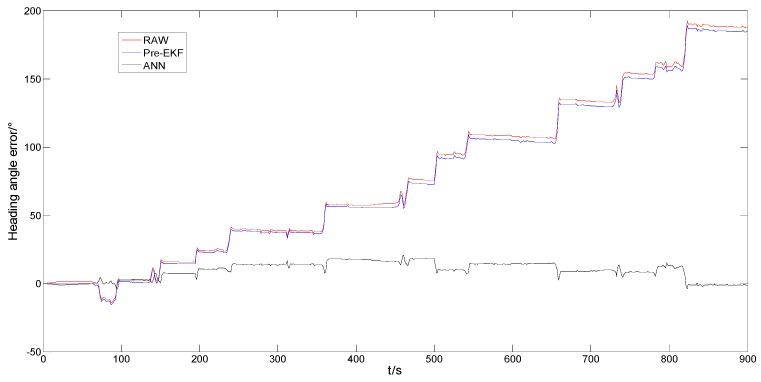
Heading angle error (rad) versus driving time (s).

Comparison of the positioning effects of the data gained from 15 min performance display data being processed by means of the neural network model in case of GNSS information being shielded and the high-precision positioning reference data is performed separately. The heading angle speed after Pre-EKF fusion is integrated with time to obtain the heading angle and then obtain the heading angle error with reference to the high-precision positioning heading angle. The relationship between the heading angle error and the driving time is the red curve in Figure 9, which indicates that the heading angle error rises rapidly and non-linearly with time (t) and it even is up to 180° at time (15 min). The blue curve in Figure 9 represents the changing rule of the original heading angle error for the sensors. Comparison of the blue and red curves indicates that the original heading angle errors may not be corrected by combining the Pre-EKF fusion. The relationship between the heading angle errors corrected by means of the BP neural network training algorithm and the driving time is the black curve in Figure 9. The heading angle error falls from the original 180° to 15° after processing. 

There are four kinds of data: (1) original data from ABS sensors, (2) data processed by the Pre-EKF algorithm based on the kinematics analysis, (3) data processed by means of the BP neural network model, and (4) the high-precision reference data from the Beidou differential positioning system.

**Figure 10:**
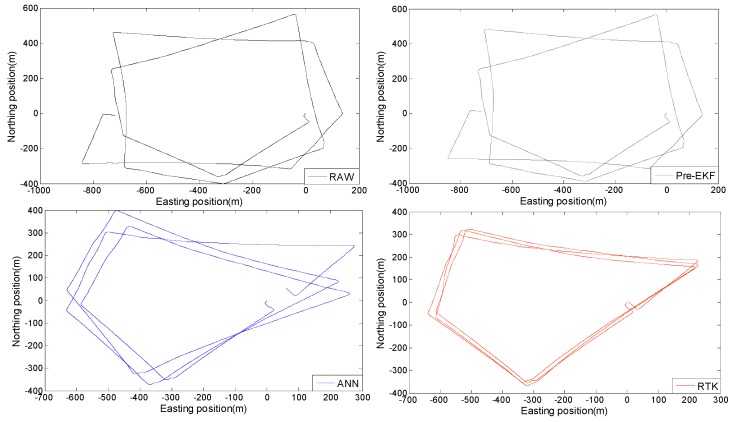
Positioning trajectories based on four kinds of data.

The above four kinds of data are marked as RAW, Pre-EKF, ANN and RTK, respectively. Their corresponding positioning trajectories are shown in Figure 10. Except for the trajectories of RTK, the other three trajectories are calculated by the heading algorithm. Analysis of the results shown in Figure 10 indicates that the Pre-EKF positioning trajectory is slightly corrected based on the RAW positioning trajectory but they are significantly different from the RTK positioning trajectory, but they deviate from the normal trajectory after the test vehicle began to turn and they may only be utilized for positioning the straight line trajectory in case of failure of the GNSS information, however, the ANN positioning trajectories are significantly improved with reference to the RAW and Pre-EKF positioning trajectories. Figure 10 indicates that ABS data may be corrected by the BP neural network model in case of failure of the GNSS information so that reliable positioning may be achieved within 15 min.

## 6. Conclusions and Outlook

In this paper, aiming at solving the problem of high noise of chip level GNSS information receiving modules in T-Boxes and being unable to locate vehicles in GNSS information failure situations due to multi-path effect during driving, a method based on ABS and GNSS information fusion positioning is proposed. By combining the collected vehicle ABS data and effective GNSS information, based on the dual extended Kalman filter algorithm, the positioning error range of the GNSS information receiving module can be reduced from 40 m to 18 m, while the error range is concentrated within 5 m. In case of GNSS information failure, the corrected BP neural network is used to modify the heading angular velocity in the ABS data. The heading angle error will not increase cumulatively with time, and reliable positioning can be realized for a long time in case of GNSS information failure.

As for the outlook of this article, firstly, when GNSS positioning is valid, but the noise of the GNSS information is too large the EKF algorithm cannot effectively reduce the error, requiring good diagnostic mechanisms for these noise GNSS information situations. Secondly, to obtain a better Q and R value in the EKF algorithm, a real-time optimization algorithm is needed to optimize Q and R based on the input.

## Figures and Tables

**Table 1:** Nomenclature related to the method.

Parameters		Parameters	
*u*	vehicle speed	*γ*	heading angle speed
*x_lo_*	longitude of BDS	*y_la_*	latitude of BDS
*x_rtk_*	longitude of RTK	*y_rtk_*	latitude of RTK
*θ*	heading angle	*PS*	positioning valid status
$\psi_{abs}$	rotation angle of the steering wheel	$\gamma_{abs}$	heading angle speed
$u_{fl}$	speed of left front-wheel	$u_{fr}$	speed of right front-wheel
$u_{rl}$	speed of left rear-wheel	$u_{rr}$	speed of right rear-wheel
$u_{k1}$	vehicle speed	$\gamma_{k1}$	heading angle speed
$\lambda_{k1}$	tangent value of front-wheel steering angle		
$x_{k2}$	relative latitude-conversion	$y_{k2}$	relative longitude-conversion
$\theta_{k2}$	heading angle	$u_{k2}$	vehicle speed
$\gamma_{k2}$	heading angle speed	Δγ	heading angle speed error

**Table 2:** Nomenclature related to the structure of the car.

Parameters		Parameters	
α	Front-wheel virtual steering angle	λ	Tangent value of front-wheel steering angle
*l*	Vehicle wheelbase	*r*	Steering radius of the vehicle
*b_f_*	Front-wheel track	*b_r_*	Rear-wheel track
$\alpha_{f}$	Deflection angle of the left front-wheel	$\alpha_{r}$	Deflection angle of the right front-wheel
*r_fl_*	Left front-wheel	*r_fr_*	Right front-wheel
*r_rl_*	Left rear-wheel	*r_rr_*	Right rear-wheel

**Table 3:** The basic parameters of the test vehicle.

Parameters	Values
Front track	1.496 m
Rear track	1.490 m
Wheelbase	2.550 m
Test speed	~40 km/h
